# A deep learning model for classifying left ventricular enlargement for both transthoracic echocardiograms and handheld cardiac ultrasound

**DOI:** 10.1093/ehjimp/qyaf049

**Published:** 2025-05-09

**Authors:** Jeffrey G Malins, D M Anisuzzaman, John I Jackson, Eunjung Lee, Jwan A Naser, Jared G Bird, Paul A Friedman, Christie C Ngo, Jae K Oh, Gal Tsaban, Patricia A Pellikka, Jeremy J Thaden, Francisco Lopez-Jimenez, Zachi I Attia, Sorin V Pislaru, Garvan C Kane

**Affiliations:** Department of Cardiovascular Medicine, Mayo Clinic, 200 First St. SW, Rochester, MN 55905, USA; Department of Cardiovascular Medicine, Mayo Clinic, 200 First St. SW, Rochester, MN 55905, USA; Department of Cardiovascular Medicine, Mayo Clinic, 200 First St. SW, Rochester, MN 55905, USA; Department of Cardiovascular Medicine, Mayo Clinic, 200 First St. SW, Rochester, MN 55905, USA; Department of Cardiovascular Medicine, Mayo Clinic, 200 First St. SW, Rochester, MN 55905, USA; Department of Cardiovascular Medicine, Mayo Clinic, 200 First St. SW, Rochester, MN 55905, USA; Department of Cardiovascular Medicine, Mayo Clinic, 200 First St. SW, Rochester, MN 55905, USA; Department of Cardiovascular Medicine, Mayo Clinic, 200 First St. SW, Rochester, MN 55905, USA; Department of Cardiovascular Medicine, Mayo Clinic, 200 First St. SW, Rochester, MN 55905, USA; Department of Cardiovascular Medicine, Mayo Clinic, 200 First St. SW, Rochester, MN 55905, USA; Department of Cardiovascular Medicine, Mayo Clinic, 200 First St. SW, Rochester, MN 55905, USA; Department of Cardiovascular Medicine, Mayo Clinic, 200 First St. SW, Rochester, MN 55905, USA; Department of Cardiovascular Medicine, Mayo Clinic, 200 First St. SW, Rochester, MN 55905, USA; Department of Cardiovascular Medicine, Mayo Clinic, 200 First St. SW, Rochester, MN 55905, USA; Department of Cardiovascular Medicine, Mayo Clinic, 200 First St. SW, Rochester, MN 55905, USA; Department of Cardiovascular Medicine, Mayo Clinic, 200 First St. SW, Rochester, MN 55905, USA

**Keywords:** artificial intelligence, deep learning, transthoracic echocardiography, handheld cardiac ultrasound, point-of-care ultrasound, left ventricular enlargement

## Abstract

**Aims:**

To develop a deep learning model that: (i) utilizes transthoracic echocardiography (TTE) clips to detect left ventricular (LV) enlargement without being provided information regarding a patient’s sex and body size; and (ii) can be accurately applied to clips acquired using either standard comprehensive TTE or handheld cardiac ultrasound (HCU).

**Methods and results:**

Using retrospective TTE data (training: 8722 patients; internal validation: 468 patients), we developed a deep learning model that estimates a patient’s end-diastolic LV volume (indexed to body surface area and normalized across the sexes), and then thresholds this estimate to perform the following classifications: (1) normally sized LV vs. ≥ mild LV enlargement; (2) normal/mildly enlarged LV vs. ≥ moderate LV enlargement. For retrospective datasets, the model showed strong performance in TTE across three geographically distinct locations (Minnesota and Wisconsin: 1082 patients, AUC = 0.925 and 0.953 for classifications 1 and 2, respectively; Arizona: 1475 patients, AUC = 0.935 and 0.969; and Florida: 1481 patients, AUC = 0.934 and 0.970). Additionally, performance was strong for both TTE and HCU clips collected from a prospective cohort of 410 patients who underwent HCU immediately following TTE (TTE: AUC = 0.925 and 0.971; HCU: AUC = 0.874 and 0.902, for classifications 1 and 2, respectively).

**Conclusion:**

An automated deep learning model applied to TTE or HCU images accurately categorizes LV volumes. These results lay a foundation for future work aimed at optimizing clinical outcomes for heart failure patients by enabling early detection of LV enlargement across various point-of-care settings.

## Graphical Abstract

**Graphical Abstract:**
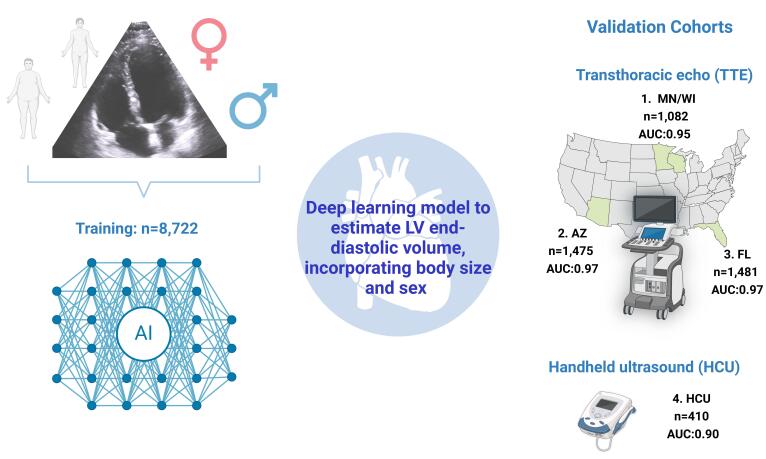
Created in BioRender. Kane, G. (2025) https://BioRender.com/c48z060.

## Introduction

The presence and severity of left ventricular (LV) enlargement (or dilation) is associated with patient morbidity and mortality,^1,2^ and is a critical marker and indicator of risk across a spectrum of cardiac diseases including heart failure,^3^ arrhythmias,^4^ and sudden cardiac death.^4,5^ Furthermore, detecting the presence and severity of LV enlargement plays an important role in risk stratification and timing of intervention in valvular heart disease.^6,7^ The use of artificial intelligence (AI) models coupled with the use of handheld cardiac ultrasound (HCU) in point-of-care settings (point-of-care ultrasound) can facilitate early and rapid detection of LV enlargement, which is critical for optimizing clinical outcomes. However, for an AI model to perform well in point-of-care settings, it should be designed with HCU in mind.^8,9^

Recently, our team developed an AI framework to estimate left ventricular ejection fraction (LVEF) from any echocardiographic view containing the LV.^10,11^ Importantly, this framework is fully end-to-end and does not require any manual clip selection by the user of the algorithm.^12^ Furthermore, the model avoids the use of image segmentation, which has been shown to be particularly error-prone for HCU images.^13^

In the current study, by adapting the framework, we already have in place for LVEF, we trained and evaluated a model using transfer learning techniques to classify a patient as having a normally sized, mildly enlarged, or moderately (or greater) enlarged indexed LV size (end-diastolic volume) based on one or more input echo video clips. Because our goal was to have this model be suitable for HCU in addition to performing well for standard comprehensive transthoracic echocardiography (TTE), the current model is similar to our previous LVEF model in that it does not segment images. Furthermore, rather than estimating raw volumes, the current algorithm instead estimates *indexed* LV size and uses normalized LV volumes across the sexes. This procedure minimizes manual inputs on the part of the operator—who would otherwise have to input a patient’s body surface area and sex to arrive at a decision regarding LV enlargement—allowing for maximal utility in a point-of-care setting.^14^

By developing an AI model for LV enlargement that performs well for both TTE and HCU data, our aim was to build a tool that offers numerous clinical benefits, including limiting inter-observer variability in measurement of LV enlargement, facilitating patient screening and triage, and streamlining clinical decision-making.^15^

## Methods

### Data acquisition and selection

The model development dataset consisted entirely of TTE patients from various community, outpatient, and hospital-based echocardiography laboratories at Mayo Clinic Rochester and Mayo Clinic Health System sites across Minnesota and Wisconsin (one exam per patient from 8722 patients total, with echocardiogram study dates between 1 February 2016 and 31 December 2021), and was an amalgamation of two distinct cohorts: one enriched for the middle-to-lower end of the LVEF distribution, and a set of healthy control patients. These two cohorts were used in our previously developed LVEF estimation model,^10,11^ but the current training set contains the subset of patients from these two cohorts whose report contained the ground truth measurement that was used for LV size (end-diastolic LV volume measured by the biplane 2D method of disks and indexed by body surface area). A subset of patients from the internal validation dataset for the previous LVEF model (with the ground truth LV volume measurement used in the current study) was used for model selection and hyperparameter tuning (*N* = 468 patients).

Model evaluation was performed using four datasets as follows: (i) a holdout testing dataset of 1082 patients from Mayo Clinic Rochester and Mayo Clinic Health System sites across Minnesota and Wisconsin, drawn from the cohort of patients enriched for mid-to-low LVEF that was used for training (but not overlapping with any of the patients whose data were used for training); (ii) an ‘all-comers’ sample of randomly selected patients with TTE exams collected at the Mayo Clinic site in Scottsdale, Arizona (1475 patients) between 1 January 2022 and 29 February 2024; (iii) an ‘all-comers’ sample of randomly selected patients with TTE exams collected at the Mayo Clinic site in Jacksonville, Florida (1481 patients) between 1 January 2022 and 29 February 2024; (iv) a prospective cohort of 410 patients who had TTE and HCU data collected during the same session. These patients visited Mayo Clinic Rochester for a clinically indicated TTE exam between 1 November 2022 and 30 September 2023. For patients who provided verbal consent for additional clips to be collected for research purposes, the following five HCU 2D video clips were collected by a trained research sonographer using a Philips Lumify handheld device: parasternal long axis (PLAX), parasternal short axis, apical 4 (A4C), apical 3 (A3C), and apical 2 chamber (A2C) views.

Information regarding study inclusion and exclusion criteria is provided in the Supplementary material. All study procedures were approved by the Mayo Clinic Institutional Review Board.

### Model design

We used our previously developed LVEF estimation model^10,11^ as a base model and applied the transfer learning technique. This model uses the S3D architecture.^16^ Pre-trained model weights from the LVEF estimation model were loaded prior to initializing training, and no layers were frozen.

Although we trained a regression model, meaning that the model output was a continuous estimate of indexed LV volume, model performance was evaluated based on classification decisions regarding clinically meaningful thresholds of LV enlargement. Therefore, each patient was grouped into one of three categories based on their ground truth measurement for indexed LV volume: normal (29–75 mL/m^2^ for female patients and 37–84 mL/m^2^ for male patients), mildly enlarged (76–87 mL/m^2^ for female patients and 85–96 mL/m^2^ for male patients), and moderately or severely enlarged (≥88 mL/m^2^ for female patients and ≥97 mL/m^2^ for male patients).^9^ The model aimed to assess LV volume irrespective of a patient’s sex. Since LV enlargement thresholds are sex-specific, values used for model training were standardized across the sexes by adding nine to the ground truth LV volume for female patients, as the recommended classification category thresholds are nine higher for males than females. Ground truth measurements were those reported by a staff echocardiography physician with advanced training in echocardiography (level III^17^).

Model input consisted of a single DICOM clip that met the following criteria: B-mode clip, at least 48 frames, and not zoomed. We selected 24 frames to constitute a single input video, which was taken from a fixed-length segment, skipping every other frame. The clip was also required to belong to the following set of view categories: A2C, A3C, A4C, and PLAX (what we refer to as the set of ‘valid’ class clips that contain the left ventricle), which corresponded to having an inference score of at least 0.5 for any one of these four view categories as defined using our previously developed view classifier.^18^ If multiple clips met these criteria per patient, all of them were considered, even if they were incomplete, so long as they had the minimum number of frames. Prior to model presentation, clips were pre-processed using the set of routines described in the Supplementary material.

Model output consisted of a floating-point estimate of indexed LV volume based on an input video clip, which was then used to classify whether or not the LV is enlarged for the patient from whom the clip was collected. If more than one video clip was selected for analysis for a patient exam, each video clip was provided as input to the model and an output value derived for each, and then these scores were averaged. The average was then thresholded to arrive at a decision regarding LV enlargement for the patient exam.

**Figure 1:**
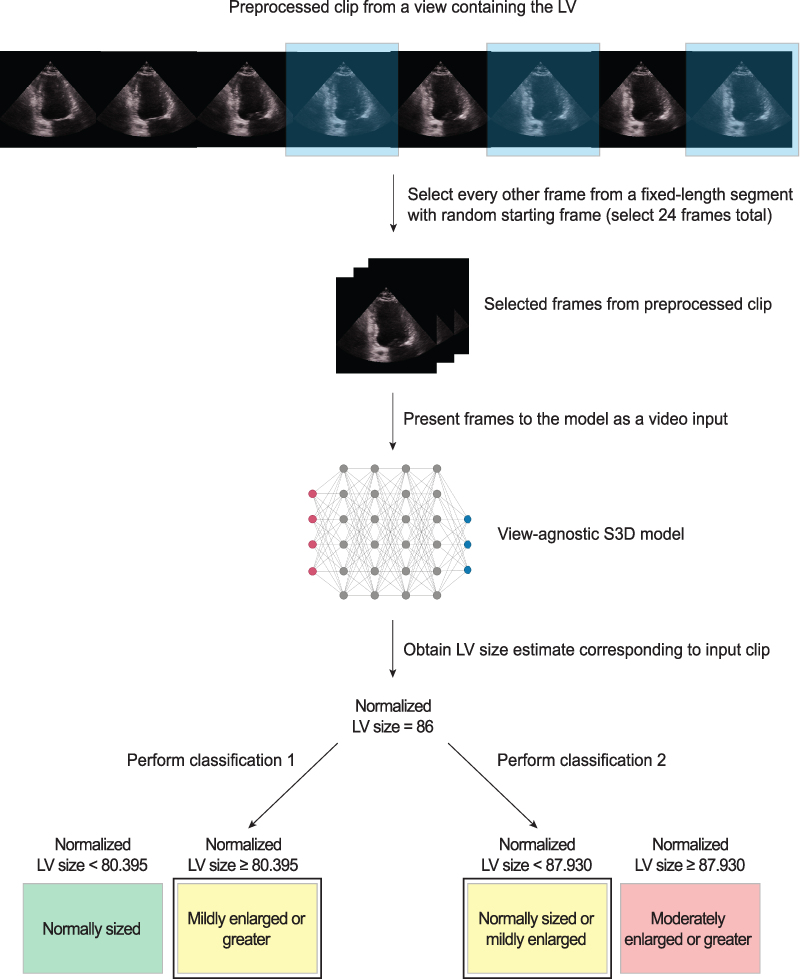
Schematic of the deep learning workflow for classification of LV enlargement. Note that the sample pre-processed clip at the top of the figure would be a TTE or HCU clip that our view classifier identified as A2C, A3C, A4C, or PLAX (in this example, A2C). The frames from this clip would be pre-processed using routines that first isolate the imaging sector and mask it, and then subsequently remove portions of the image surrounding the mask (i.e. text, labels, and so on) prior to the workflow shown here. Note that the LV size estimate is hypothetical for illustrative purposes. The icon used to denote the deep learning network was taken from BioRender.com.

A schematic of the workflow for the deep learning model is provided in *Figure 1*.

### Model training procedure

For model training, we randomly selected two windows from each training video. The rationale for the sliding window technique was that this method allowed the model to be more robust to temporal variability in the cardiac cycle. The model was trained on 85 524 clips from 8722 patients, with two windows per clip (171 048 clip segments total). Random image augmentation (described in the Supplementary material) was used to further diversify the training set.

The model was trained for 100 epochs for 2.5 days using four Tesla A100 GPUs, each with 40GB of memory. The root mean square error loss function was used during model training (more details are provided in the Supplementary material regarding how patient-wise loss was computed for the tuning dataset). A batch size of 40 was used, divided equally across four GPUs using the data parallelization technique. We used a learning rate scheduler (starting at 0.001 and multiplied by 0.1 every 30 epochs) and optimization was performed using the Adam algorithm with a weight decay factor of 0.00001. We also used the mixed precision technique for faster and more balanced model training.

### Statistics

To evaluate model performance, optimal cut-point thresholds were computed. These thresholds balanced sensitivity and specificity in the tuning dataset for each of the classification decisions: (i) a normally sized LV vs. a mildly enlarged LV (or greater); (ii) a normally sized or mildly enlarged LV vs. a moderately enlarged LV (or greater). We then applied these thresholds (80.395 and 87.930 for the two classification decisions, respectively) to evaluate model performance for the testing datasets. Note that each classification decision only required a single threshold because indexed values had been standardized across sexes. We used the fast implementation of DeLong’s method^19^ to compute 95% CIs for AUCs.

## Results

### Sample characteristics

*Table 1* provides information regarding the model development and evaluation cohorts, and Supplementary data online, *Figure S1* illustrates the distribution of ground truth values for the model training cohort. As shown in *Table 1*, the model development and evaluation cohorts were similar in terms of patient demographics and comorbidities. However, because the Arizona and Florida testing datasets were completely random samples, whereas the training and tuning datasets and the holdout testing dataset from Minnesota and Wisconsin were enriched for those with mid-to-low LVEF (many of whom also had mild or greater LV enlargement), the proportion of patients with a normally sized LV was greater in the Arizona and Florida cohorts than it was in the other cohorts.

**Table 1:** Demographic and clinical characteristics of the patient cohorts for model development and model evaluation

	Model development		Model evaluation				
	Training	Tuning	Minnesota–Wisconsin	Arizona	Florida	Simult. TTE and HCU	
Number of studies	8722	468	1082	1475	1481	410	
Patient demographics							
Age on study date (years)							
Mean ± standard deviation	64 ± 16	64 ± 15	66 ± 16	68 ± 15	65 ± 15	63 ± 16	
Range	18–100	18–97	18–104	18–102	18–96	18–93	
Sex (% of cohort)							
Female	43.14	39.96	40.57	40.47	45.17	36.83	
Male	56.86	60.04	59.43	59.53	54.83	63.17	
Race and ethnicity (% of cohort)							
NH American Indian/Alaska Native	0.38		0.37	1.76	0.20	0.73	
NH Asian	1.27	1.28	0.83	3.59	2.03	1.95	
NH Black/African American	1.91	2.14	2.40	3.12	8.85	2.20	
Hispanic/Latino	1.91	1.50	2.96	9.22	5.81	1.46	
NH Native Hawaiian/Other Pacific Islander	0.07		0.18	0.41	0.27		
NH White	90.48	92.31	89.28	79.19	80.22	90.73	
Other or unknown	3.96	2.78	3.97	2.71	2.57	2.93	
Body mass index, kg/m^2^							
Mean ± standard deviation	29 ± 7	28 ± 5	29 ± 7	28 ± 6	29 ± 6	28 ± 5	
Range	12–85	17–52	15–70	13–56	15–63	15–47	
Body mass index (% of cohort)							
BMI < 25	25.49	25.00	24.41	30.24	27.01	26.34	
25 ≤ BMI < 30	30.14	35.04	27.36	33.15	32.41	40.73	
BMI ≥ 30	34.50	34.40	37.62	33.56	34.37	29.02	
Missing measurement	9.87	5.56	10.81	3.05	6.21	3.90	
Comorbidities (% positive)							
Hypertension	59.67	68.59	69.69	68.68	65.63	54.88	
Atrial fibrillation	27.76	30.34	33.46	35.46	34.37	32.93	
Hyperlipidaemia	58.01	59.19	62.75	62.98	60.09	56.59	
Congestive heart failure	39.25	44.44	47.69	39.19	34.64	45.37	
Myocardial infarction	21.20	20.51	26.16	20.00	17.29	11.95	
Valvular disease	42.65	54.70	49.82	74.24	62.05	63.66	
Peripheral vascular disorders	37.56	45.73	42.98	44.75	37.81	48.05	
Chronic pulmonary disease	32.55	30.56	34.38	24.88	25.32	21.71	
Diabetes mellitus	24.52	25.21	29.30	24.34	26.06	15.85	
Renal failure	25.91	26.92	32.99	32.34	28.97	24.15	
Liver disease	17.14	14.53	17.56	19.59	23.36	14.63	
Echocardiography							
LV end-diastolic volume (indexed)—continuous measurement (mL/m^2^)^a^							
Mean ± standard deviation	71 ± 25	79 ± 24	70 ± 26	56 ± 19	59 ± 20	74 ± 20	
Range	29–409	29–191	26–354	26–189	26–252	32–172	
LV end-diastolic volume (indexed)—category							
Normally sized (or smaller)^b^	75.30	56.32	76.99	92.00	89.45	75.12	
Mildly enlarged	11.22	21.20	10.07	3.66	5.47	12.68	
Moderately enlarged or greater	13.48	22.48	12.94	4.34	5.06	12.20	
LV ejection fraction							
Mean ± standard deviation	54 ± 13	53 ± 12	52 ± 13	59 ± 10	60 ± 10	56 ± 11	
Range	7–80	15–73	10–79	12–79	6–82	17–75	
A4C orientation (% of cohort)						TTE	HCU
Left heart on left of image	78.19	86.32	72.18	100	100	100	100
Right heart on left of image	21.81	13.68	27.87				
Equipment manufacturer (% cohort)						TTE	HCU
GE Healthcare	89.09	95.94	87.43	99.46	97.23	100	
Philips Medical Systems	10.91	4.06	12.57	0.54	2.77		100

Sex as well as race and ethnicity were self-reported and obtained from each patient’s medical record. Comorbidities were ascertained using the *comorbidity* package in R^24^ based on ICD-9 or ICD-10 codes noted in the patient’s medical record up to and including the echocardiographic study date. The LV end-diastolic volume is indexed to body surface area (mL/m^2^).

Simult., simultaneously collected; TTE, transthoracic echocardiography; HCU, handheld cardiac ultrasound; NH, non-Hispanic; A4C, apical 4-chamber.

^a^There were six patients in the training cohort and one patient in the tuning cohort whose indexed LV end-diastolic volume contained an error in the report (based on an incorrectly transcribed height or weight measurement). The mean, standard deviation, and range for this measurement are shown with these values removed, and proportions in each category are computed for the remaining portion of the cohort.

^b^Eighteen patients in the Minnesota-Wisconsin cohort, 55 patients in the Arizona cohort, and 26 patients in the Florida cohort had an indexed LV end-diastolic volume measurement that was below the sex-specific thresholds to be considered normally sized. For the purposes of model evaluation, these patients were considered as having a normally sized LV for their ground truth measurement.

### Model performance

**Figure 2:**
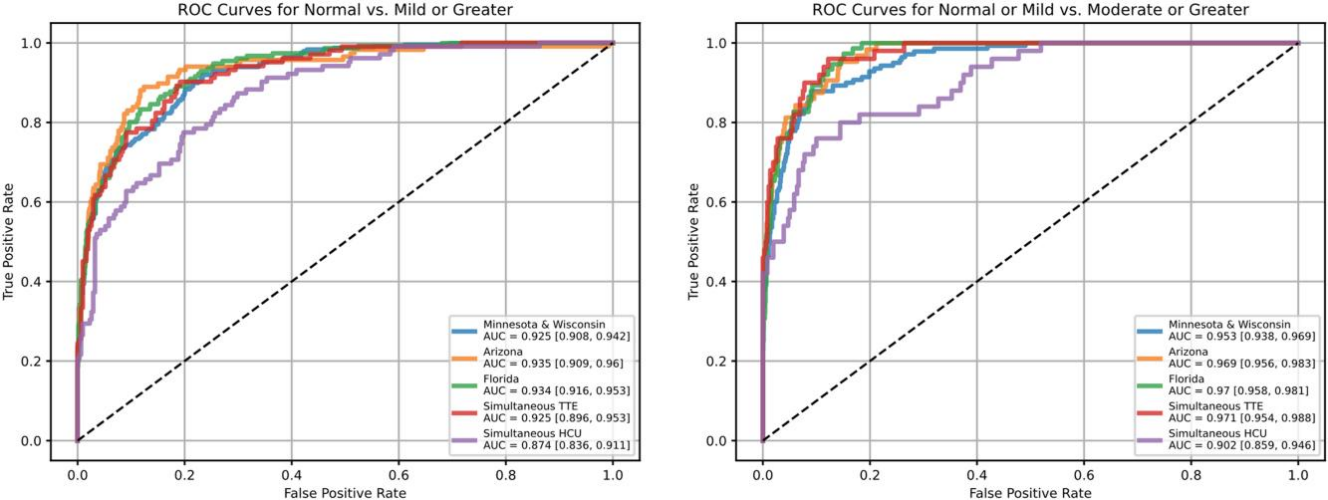
ROC curves for each of the model evaluation datasets for the classification decision of a normally sized LV vs. ≥ mild LV enlargement (left plot), or a normal/mildly enlarged LV vs. ≥ moderate LV enlargement (right plot). The values in square brackets are 95% confidence intervals of the AUC. AUC, area under the operating curve; TTE, transthoracic echocardiography; HCU, handheld cardiac ultrasound.

Receiver-operator-curves (ROC) curves for all cohorts are displayed in *Figure 2* and overall performance metrics are summarized in *Table 2*. It should be noted that when comparing performance for TTE and HCU clips in the simultaneous TTE-HCU dataset, we selected one clip per view for the TTE dataset (i.e. the clip with the highest score from our view classifier for each view) and took the average estimate across clips. This was done to enable a direct comparison with the HCU dataset, for which only one clip was collected for each view. In contrast, for the retrospective TTE datasets, we averaged results across all clips containing the left ventricle (not just one clip per view). In addition to evaluating model performance using the average estimate across all views, Supplementary data online, *Figure S2* details model performance for each view individually as well as combinations of individual views.

**Table 2:** Performance metrics for LV enlargement classification decisions across patient cohorts

Classification	Metric	MN–WI	AZ	FL	Simult. TTE	Simult. HCU
Normal vs. mild or greater	Sensitivity	0.767	0.839	0.801	0.853	0.588
	Specificity	0.879	0.894	0.897	0.831	0.919
	PPV	0.654	0.407	0.477	0.626	0.706
	NPV	0.927	0.985	0.975	0.945	0.871
	AUC (95% CI)	0.925 (0.908, 0.942)	0.935 (0.909, 0.960)	0.934 (0.916, 0.953)	0.925 (0.896, 0.953)	0.874 (0.836, 0.911)
Normal or mild vs. moderate or greater	Sensitivity	0.836	0.844	0.827	0.900	0.580
	Specificity	0.924	0.939	0.940	0.914	0.942
	PPV	0.619	0.386	0.422	0.592	0.580
	NPV	0.974	0.993	0.990	0.985	0.942
	AUC (95% CI)	0.953 (0.938, 0.969)	0.969 (0.956, 0.983)	0.970 (0.958, 0.981)	0.971 (0.954, 0.988)	0.902 (0.859, 0.946)

MN–WI, Minnesota–Wisconsin; AZ, Arizona; FL, Florida; PPV, positive predictive value; NPV, negative predictive value; AUC, area under the curve; CI, confidence interval.

As shown in *Figure 2*, model performance was consistently strong across all retrospective TTE cohorts for both classification decisions, although slightly inferior for the classification of normal vs. mild or greater LV enlargement (Minnesota and Wisconsin: AUC = 0.925; Arizona: AUC = 0.935; Florida: AUC = 0.934) than it was for the decision of normal or mild vs. moderate or greater LV enlargement (Minnesota and Wisconsin: AUC = 0.953; Arizona: AUC = 0.969; Florida: AUC = 0.970). In addition, performance was slightly inferior for the HCU data in the prospective cohort than it was for TTE data collected in the same set of patients (TTE: AUC = 0.925 and 0.971; HCU: AUC = 0.874 and 0.902, for the two classification decisions, respectively), but nevertheless still strong.

### Sensitivity analyses for patient subgroups according to patient sex and BMI

*Table 3* details AUC values for the model evaluation cohorts according to subgroups for patient sex and BMI. As shown in *Table 3*, performance was strong for all subgroups (AUC ≥ 0.875 for all TTE datasets and AUC ≥ 0.813 for HCU). In some cohorts, model performance was slightly inferior for female patients compared with male patients for the classification of normally sized vs. mildly enlarged or greater. However, this pattern was not consistent across all cohorts, and furthermore was reversed for the classification of normally sized or mildly enlarged vs. moderately enlarged or greater. For BMI, model performance showed consistent trends across cohorts for each of the three subgroups.

**Table 3:** AUCs (with 95% confidence intervals) for LV enlargement classification decisions across subgroups according to patient sex and body mass index

Classification	Subgroup	MN–WI	AZ	FL	Simult. TTE	Simult. HCU
Normal vs. mild or greater	Female	0.940 (0.910, 0.969)	0.875 (0.792, 0.959)	0.931 (0.894, 0.967)	0.908 (0.851, 0.965)	0.813 (0.715, 0.911)
	Male	0.913 (0.891, 0.936)	0.953 (0.934, 0.972)	0.932 (0.910, 0.953)	0.929 (0.897, 0.962)	0.905 (0.867, 0.944)
	BMI < 25	0.901 (0.859, 0.942)	0.947 (0.910, 0.983)	0.904 (0.857, 0.951)	0.907 (0.841, 0.973)	0.877 (0.805, 0.949)
	25 ≤ BMI < 30	0.929 (0.899, 0.960)	0.953 (0.927, 0.979)	0.949 (0.928, 0.971)	0.932 (0.892, 0.972)	0.899 (0.848, 0.950)
	BMI ≥ 30	0.941 (0.915, 0.966)	0.931 (0.886, 0.975)	0.948 (0.919, 0.977)	0.925 (0.875, 0.976)	0.862 (0.789, 0.935)
Normal or mild vs. moderate or greater	Female	0.968 (0.948, 0.988)	0.958 (0.922, 0.993)	0.985 (0.972, 0.997)	0.982 (0.964, 1)	0.938 (0.848, 1)
	Male	0.944 (0.923, 0.965)	0.970 (0.955, 0.985)	0.956 (0.937, 0.974)	0.966 (0.942, 0.989)	0.897 (0.848, 0.946)
	BMI < 25	0.942 (0.907, 0.976)	0.973 (0.950, 0.996)	0.960 (0.935, 0.985)	0.953 (0.908, 0.998)	0.912 (0.825, 0.999)
	25 ≤ BMI < 30	0.953 (0.923, 0.983)	0.963 (0.936, 0.990)	0.968 (0.946, 0.989)	0.988 (0.974, 1)	0.890 (0.811, 0.970)
	BMI ≥ 30	0.958 (0.935, 0.980)	0.970 (0.946, 0.993)	0.978 (0.960, 0.996)	0.961 (0.924, 0.998)	0.925 (0.864, 0.986)

MN–WI, Minnesota–Wisconsin; AZ, Arizona; FL, Florida; Simult., simultaneously collected; BMI, body mass index in kg/m^2^.

## Discussion

The aim of the current study was to develop a deep learning model to detect the presence of LV enlargement using echocardiographic clips collected using either standard comprehensive TTE or HCU and also incorporating the patient’s sex and body size. To do this, we used a large training set of retrospective TTE video clips to train a model that classifies a patient as having a normally sized, mildly enlarged, or moderately enlarged (or greater) indexed LV based on one or more input echo video clips. We then evaluated the model on separate sets of TTE data as well as a prospective dataset of HCU images (with simultaneous TTE as reference).

There were several key findings. First, the model showed strong performance across all the retrospective TTE testing datasets. These findings are in line with previous work that has shown success in estimating end-diastolic and end-systolic volumes of the LV without requiring segmentation of images,^20^ and differs from other approaches that either require segmentation of the LV^21–23^ or estimation of LV diameter that is then used to compute LV volume.^1^ Second, when TTE and HCU clips from the same set of patients were presented to the model and outputs compared, the model performed well for HCU clips, albeit not quite as strongly as for TTE clips. This performance is notable because the model estimated indexed end-diastolic volume without being explicitly given information about the timing of the cardiac cycle, which is particularly useful for HCU data that does not include ECG-gating. Similar to our previous work with LVEF, the current results suggest that TTE data (which is generally more plentiful) can be leveraged to train AI models that perform well for HCU.^9^ Finally, sensitivity analyses for patient subgroups stratified by patient sex and BMI revealed that the model performed well across various subgroups.

These results have several important clinical implications. First, early detection of LV dilation could aid in risk stratification for heart failure,^3^ arrhythmia,^4^ valvular heart disease,^6,7^ and sudden cardiac death,^5^ and furthermore can inform the timing of intervention in valvular heart disease.^6,7^ Embedding the AI algorithm for use in HCU could enable screening populations who are not currently receiving formal echocardiographic exams and who may be at risk for these cardiac diseases, such as community samples as well as patients in primary care environments or emergency departments. Second, rather than estimating a continuous volume, which a provider would then have to interpret using various thresholds according to the patient’s sex and body surface area, the current model provides a classification decision regarding LV enlargement that does not require an operator to input the patient’s sex or body surface area. This could be particularly useful in point-of-care settings in which providers need to make rapid decisions regarding patient triage—such as whether a patient should be recommended to receive a full comprehensive echocardiographic exam—and obtaining and inputting patient sex and height and weight could be an impediment to AI model use.^14^

With that said, the current results should be considered in light of several critical limitations. First, the current set of HCU clips were collected by experienced sonographers in a controlled environment, and we also note the presence of a selection bias in the simultaneously collected TTE-HCU cohort, as the HCU data were collected from patients who had been recommended for a clinically indicated formal TTE exam. To address this, future work should validate the model using data collected by operators with varying levels of experience and in a variety of settings. Second, although the model performed well across various subgroups, future research should focus more systematically on specific comorbidities that are known to differ in prevalence according to factors such as patient sex and BMI, and furthermore, the model should be validated with cohorts with greater diversity of race and ethnicity than the current cohorts. Third, we acknowledge that although the model evaluation cohorts included different geographic sites as well as a mixture of inpatient vs. outpatient settings and small-community practices (i.e. many of the Mayo Clinic Health System sites), the data were all collected in the same healthcare system, and future work should focus on validating the model in external clinical environments.

Nevertheless, the current set of findings indicate the AI model shows initial promise in detecting LV enlargement across a variety of patients and suggests that the current approach is viable for use with both TTE and HCU data. Together, these results lay a foundation for future work that could optimize clinical outcomes for patients with heart failure by allowing for early detection of LV enlargement in point-of-care settings. Next steps for clinical implementation will include validation using external samples with diversity in patient demographics, clinical environments, and operator experience, as well as rigorous investigation into how best to embed the model into clinical workflows to maximize its clinical utility.

## Supplementary Material

qyaf049_Supplementary_Data

## Data Availability

All requests for raw and analysed data and related materials, excluding programming code, will be reviewed by the Mayo Clinic legal department and Mayo Clinic Ventures to verify whether the request is subject to intellectual property obligations and to ensure patient confidentiality. Any data and materials that can be shared will be released via a Material Transfer Agreement. Programming code related to the PyTorch model specification will be made available under the GNU General Public License version 3 upon request to the corresponding author.

## Lead author biography

Jeffrey Malins, PhD, is a Principal Data Science Analyst working in the Department of Cardiovascular Medicine, Mayo Clinic. His research focuses on applications of artificial intelligence in cardiovascular imaging, particularly echocardiography. Prior to joining Mayo Clinic, he worked in the fields of neuroimaging and psychology.

D M Anisuzzaman, PhD, is a Senior Data Science Analyst in the Department of Cardiovascular Medicine at Mayo Clinic. He develops deep learning models for cardiovascular diagnosis and prognosis from echocardiograms and free-speech voice. Previously, he applied artificial intelligence to wound care research.
