# Correction to: A deep learning model for classifying left ventricular enlargement for both transthoracic echocardiograms and handheld cardiac ultrasound

**DOI:** 10.1093/ehjimp/qyaf104

**Published:** 2025-08-14

This is a correction to: Jeffrey G Malins, D M Anisuzzaman, John I Jackson, Eunjung Lee, Jwan A Naser, Jared G Bird, Paul A Friedman, Christie C Ngo, Jae K Oh, Gal Tsaban, Patricia A Pellikka, Jeremy J Thaden, Francisco Lopez-Jimenez, Zachi I Attia, Sorin V Pislaru, Garvan C Kane, A deep learning model for classifying left ventricular enlargement for both transthoracic echocardiograms and handheld cardiac ultrasound, *European Heart Journal - Imaging Methods and Practice*, Volume 3, Issue 2, July 2025, qyaf049, https://doi.org/10.1093/ehjimp/qyaf049

Due to an error, this paper was incorrectly published in the Special Issue on Advancements in Diagnostic Imaging for Congenital Heart Disease, Volume 3, Issue 3. It has now been republished in Volume 3 Issue 2 of the journal.

