# Supplementary material for: A deep learning model for classifying left ventricular enlargement for both transthoracic echocardiograms and handheld cardiac ultrasound
Source: Eur Heart J Imaging Methods Pract. 2025 May 9;3(2):qyaf049. doi: 10.1093/ehjimp/qyaf049 (PMC12275095; doi:10.1093/ehjimp/qyaf049)
Supplement: qyaf049_Supplementary_Data [file qyaf049_Supplementary_Data.pdf]

A Deep Learning Model for Classifying Left Ventricular Enlargement for both Transthoracic Echocardiograms and Handheld Cardiac Ultrasound

Jeffrey G. Malins, PhD; D M Anisuzzaman, PhD; John I. Jackson, PhD; Eunjung Lee, PhD; Jwan A. Naser, MBBS; Jared G. Bird, MD; Paul A. Friedman, MD; Christie C. Ngo, BSc; Jae K. Oh, MD; Gal Tsaban, MD, PhD; Patricia A. Pellikka, MD; Jeremy J. Thaden, MD; Francisco Lopez-Jimenez, MD, MSc, MBA; Zachi I. Attia, PhD ; Sorin V. Pislaru, MD, PhD; Garvan C. Kane, MD, PhD

Supplementary Material

Table of Contents

Supplementary Methods 2

Data acquisition and selection 2

Data processing 3

Model training procedure 3

Supplementary Results 5

Figure S1: Distribution of ground truth measurements 5

Figure S2: Model performance for individual views and combinations of views 5

References 6

Supplementary Methods

Data acquisition and selection

Studies had to meet the following requirements to be included in the model development or evaluation cohorts:

1. The ground truth measurement had to be within an acceptable range, as defined by clinically relevant thresholds (1). For the training dataset, patients were excluded if their end-diastolic LV volume, measured by the biplane 2D method of disks and indexed by body surface area, was less than 29 mL/m^2 for females and less than 37 mL/m^2 for males. For model evaluation datasets, patients were excluded if their end-diastolic LV volume, measured by the biplane 2D method of disks and indexed by body surface area, was less than 25 mL/m^2, as values lower than this were considered errors.
2. A patient's previous data was not used to train our previously developed view classifier. The rationale for this was to avoid cross-contamination of samples, as the view classification deep learning model was embedded in the current deep learning framework.
3. Only one study phase (i.e., just baseline rather than baseline plus other phases such as Valsalva or inspiratory). The rationale for this is that based on how echocardiographic videos are stored in our data management platform, we could not ascertain which clips corresponded to the ground truth measurement if there was more than one study phase.
4. At least one valid-class clip with at least 48 frames (described in the 'Model design' section of the main text), as this was the minimum input for the models we developed.

Data processing

DICOM clips were preprocessed using the following set of operations. First, frames were extracted using an algorithm that isolates the imaging sector (i.e., the 'cone'-shaped portion of a B-mode image) and excludes text information and other labels outside of the imaging sector. This was done by identifying pixels whose intensity values changed over the course of videos. Following this, morphological operations and colour-based techniques were used to remove the ECG trace, with context-based filling used to replace missing pixels once the ECG was removed (*opencv* version 4.5.5 in Python). The image was then cropped to fit the imaging sector and padded with columns or rows of zeroes (depending on whether the image width was less than or greater than the height) to generate a square image 256 by 256 pixels in size.

Model training procedure

To increase model generalizability, we applied five augmentations to the training dataset: random rotation between -10 and +10 degrees, Gaussian blurring, central cropping (i.e., isolating the centre of each frame), random cropping (i.e., isolating one of the four corners of each frame), and horizontal flipping. We applied random rotation because we observed that due to variation in probe placement during data acquisition, the echocardiographic imaging sector (and cardiac anatomy) was sometimes not centred at the middle of each video frame; we applied Gaussian blurring to make the model more robust for lower quality echocardiographic videos; we applied central and corner cropping because we observed that on some videos, some portions of the heart chambers were

cropped. Gaussian blurring was performed using a 7 x 7 Gaussian kernel, and a sigma with a range from 0.1 to 0.2. Central and corner cropping isolated the centre or one of the four corners of each frame using a 180 x 180 window, with resultant images then resized back to 256 x 256 pixels. Horizontal flipping was performed so that the model would be exposed to A4C clips in left-on-left and right-on-left orientations, since much of the training data had the left-on-left orientation used in most Mayo Clinic sites, which differs from most other sites across the world.

Augmentations were performed in a batchwise fashion, with each clip having a one-sixth likelihood of either receiving no augmentation (i.e., the original data) or one of the five augmentations listed above (chosen randomly). All 24 frames within each input clip were subjected to the same augmentation. No augmentation was applied to the tuning dataset.

When computing loss for the tuning dataset, in each epoch, estimates per patient were obtained by first taking five randomly pulled clips with five windows from each clip, and then passing as input to the model 24 frames from a fixed-length segment, skipping every other frame, with the window randomly positioned within each clip. Model estimates were then averaged across all selected windows and clips for that patient. Trained models were saved, and the best model was selected (at epoch 41) based on minimum patient-wise loss for the tuning dataset.

Supplementary Results

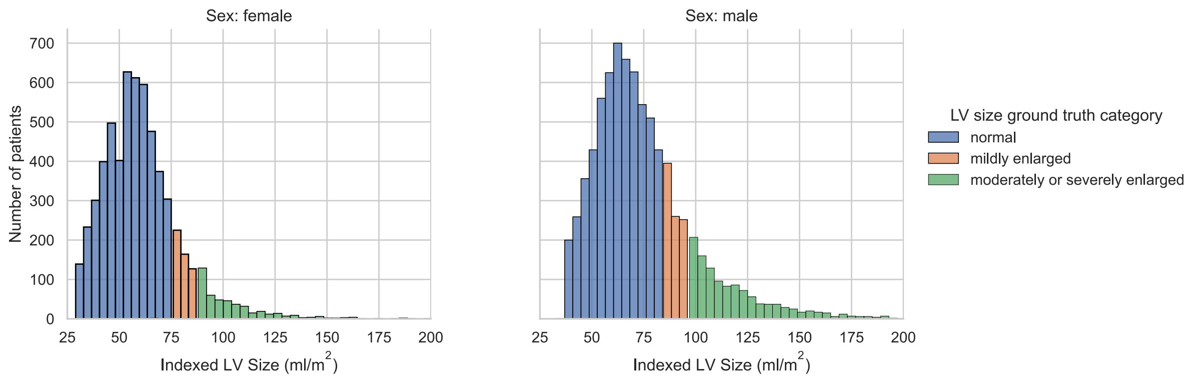

Figure S1. Distribution of ground truth measurements of indexed LV size in the training cohort (8,722 patients). Note that prior to training, measurements for female patients were normalized by adding a value of 9 (not reflected in the measurements shown in the histogram). Note also that the colour-coded categories are only shown for reference purposes and continuous values were used during training of the regression model.

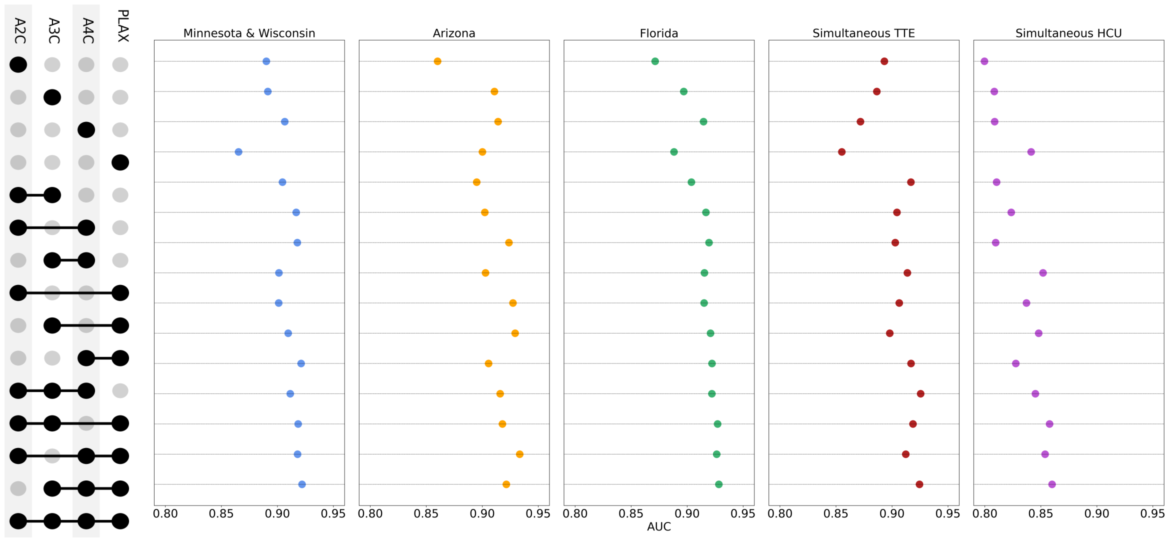

Figure S2. Model performance for individual views and combinations of views. The top four rows indicate performance for individual views (i.e., only one clip total per patient), whereas the other rows indicate combinations of views, with model performance evaluated using the average model estimate per patient across these views (with one clip per view). In all cases, the selected clip for each view had the highest score from the view classifier for that view. The legend to the left is plotted using version 0.9.0 of the *upsetplot* package (2) in Python version 3.9.20.

References

1. Asch FM, Miyoshi T, Addetia K, Citro R, Daimon M, Desale S, et al. Similarities and differences in left ventricular size and function among races and nationalities: Results of the World Alliance Societies of Echocardiography Normal Values Study. Journal of the American Society of Echocardiography. 2019;32(11):1396-406.e2.
2. Lex A, Gehlenborg N, Strobelt H, Vuillemot R, Pfister H. UpSet: Visualization of intersecting sets. IEEE Trans Vis Comput Graph. 2014;20(12):1983-92.
